# One-Step Microwave-Assisted Synthesis of MnFe_2_O_4_/rGO Nanocomposites and Their Electrochemical Properties in Supercapacitors

**DOI:** 10.1021/acsomega.4c07810

**Published:** 2025-01-27

**Authors:** Kun-Yauh Shih, Hui-Ying Tseng

**Affiliations:** Department of Applied Chemistry, National Pingtung University, Pingtung County 900391, Taiwan

## Abstract

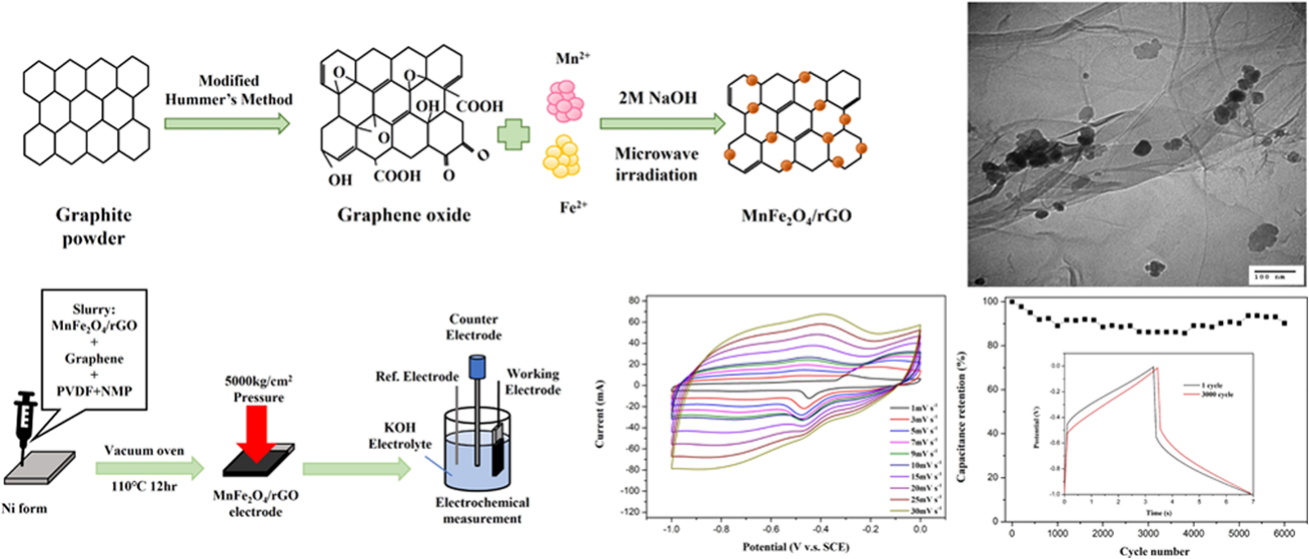

In the pursuit of energy conservation and sustainability, supercapacitors offer high power density, fast charge–discharge rates, and long cycle life, making them ideal for applications in electric vehicles and portable electronics. This study presents the synthesis of MnFe_2_O_4_/reduced graphene oxide (rGO) nanocomposites through a rapid, one-pot microwave-assisted hydrothermal method. This approach significantly reduces synthesis time relative to conventional hydrothermal techniques. The nanocomposites were structurally characterized using X-ray diffraction (XRD) and Raman spectroscopy, morphologically examined via transmission electron microscopy (TEM) and Brunauer–Emmett–Teller (BET) analysis, and chemically analyzed through Fourier-transform infrared spectroscopy (FT-IR) and thermogravimetric analysis (TGA). Electrochemical testing revealed a high specific capacitance of 196.6 F/g at 0.5 A/g, with 90.22% retention after 6000 cycles at 10 A/g. The superior performance is attributed to the synergistic effects of MnFe_2_O_4_ and rGO, enhancing charge storage and stability. These findings demonstrate the potential of microwave hydrothermal synthesis for scalable production of high-performance electrode materials for supercapacitors.

## 1 Introduction

In today’s world, efficient energy storage technologies are essential for minimizing energy wastage, promoting environmental sustainability, and reducing costs. Among various energy storage systems such as lithium batteries and hydrogen fuel cells, supercapacitors stand out due to their high power density, long cycle efficiency, and rapid charge–discharge capabilities, making them ideal for applications in hybrid electric vehicles, military equipment, and portable electronic devices.^1^

Supercapacitors are broadly categorized into electric double-layer capacitors (EDLCs), pseudocapacitors (PCs), and hybrid capacitors (SCs) based on their charge storage mechanisms.^2^ EDLCs operate through non-Faradaic reactions in which ions are adsorbed onto electrode surfaces to form an electric double layer. This process offers high energy density and long-term capacitance retention, albeit with lower energy density limitations.^3^ For instance, EDLCs have demonstrated remarkable cycling stability, with 91.3% retention after 10,000 cycles at 5 A/g.^4^ In contrast, PCs utilize Faradaic processes involving redox reactions between active materials and electrolytes, such as RuO_2_ and MnO_2_. These processes provide higher specific capacitance but often suffer from reduced cycle stability.^5,6^ Hybrid capacitors combine carbon materials like graphene with metal oxides to leverage the strengths of both EDLCs and PCs while mitigating their respective weaknesses.

Graphene, with its high surface area and superior conductivity, plays a pivotal role in enhancing supercapacitor performance. However, graphene’s tendency to aggregate and stack can limit its effectiveness, necessitating doping or structural modifications like hydrothermal reduction to produce reduced graphene oxide (rGO) with enhanced stability and conductivity.^7^ The environmental impact of active materials is also a critical consideration; while materials like RuO_2_ are effective, they are toxic and environmentally hazardous. In contrast, manganese dioxide (MnO_2_) and iron oxides (Fe_2_O_3_) such as MnFe_2_O_4_ offer promising alternatives due to their low toxicity, abundance, and excellent energy storage capabilities.^8,9^

Among metal oxides, spinel ferrites like MnFe_2_O_4_ have gained attention for their high specific capacitance, low environmental impact, and cost-effectiveness. MnFe_2_O_4_ possesses unique properties such as biocompatibility, making it suitable for diverse applications ranging from wearable devices to environmental remediation and semiconductor components.^10,11^ These materials exhibit significant recent advancements in energy storage and conversion applications.^12,13^ Moreover, they possess biocompatibility and low toxicity, making them suitable for applications in human health and wearable devices.^8,9,14^ Previous studies have demonstrated that MnFe_2_O_4_ exhibits a capacitance of 173 F/g and retains 105% efficiency after 10,000 charge/discharge cycles, highlighting its potential for robust energy storage applications.^15^

In recent years, significant efforts have been dedicated to synthesizing MnFe_2_O_4_/ rGO nanocomposites through traditional hydrothermal methods, yielding promising enhancements in the electrochemical performance of supercapacitors.^16^ Yi et al. developed high-performance supercapacitor electrode materials by employing in situ growth of Ni(OH)_2_ nanoflowers on nickel foam.^17^ Additionally, Rong et al. utilized Co(OH)_2_/rGO microfilms for flexible in-sandwich and planar microsupercapacitors, which exhibited excellent capacitance retention and flexibility.^18^

However, conventional synthesis techniques, including hydrothermal, coprecipitation, annealing,^19^ and in situ solvothermal methods,^20^ are often characterized by high temperature requirements and prolonged synthesis durations. These factors contribute to increased energy consumption and reduced overall efficiency. Additionally, an increasing number of studies are exploring modifications to the heating source or the use of alternative solvents in hydrothermal methods,^21^ with the aim of developing greener and more sustainable chemical synthesis processes. In contrast to traditional methods, microwave-assisted hydrothermal synthesis has emerged as a more efficient and scalable alternative, offering advantages such as rapid processing and uniform heating.^3^ These benefits lead to shorter nucleation and crystallization times, resulting in smaller particle sizes and higher dispersibility. Furthermore, Saloga et al.^22^ experimentally demonstrated that switching the heating source to microwave synthesis had no significant impact on the core size of the crystals; however, particle size increased with temperature.

Unlike conventional methods, microwave synthesis heats materials by inducing molecular vibrations within the container, eliminating the need for conductive heat transfer. This technique has been successfully applied in synthesizing various nanomaterials, including ZnFe_2_O_4_/rGO for methylene blue dye removal,^10^ one-step synthesis of PtNiCo/rGO electrocatalysts for direct methanol fuel cells,^23^ and Eu(OH)_3_/rGO nanoparticles with enhanced antibacterial activity against *Escherichia coli*(24) Specifically, Chernozem et al. synthesized MnFe_2_O_4_ particles using microwave-assisted hydrothermal synthesis at temperatures between 180 and 200 °C for 3 to 6 h.^25^ Recent studies have demonstrated that materials synthesized through microwave-assisted methods can significantly enhance supercapacitor performance due to their high theoretical capacitance, improved conductivity, and structural stability.^26,27^ For instance, Mo et al. successfully synthesized ZnFe_2_O_4_/rGO using a microwave hydrothermal method, achieving remarkable electrochemical characteristics suitable for supercapacitors.^3^

Microwave synthesis offers several advantages: rapid heating rates, reduced energy consumption, high product yield, superior purity, and excellent reproducibility.^22,23^ Despite these benefits, research on synthesizing MnFe_2_O_4_/rGO nanocomposites via microwave-assisted hydrothermal methods—particularly for supercapacitor electrodes—remains limited. Therefore, this study aims to synthesize MnFe_2_O_4_/rGO nanoparticles using a one-pot microwave hydrothermal method to confirm its applicability in supercapacitors while achieving a greener synthesis process. The synthesized nanocomposites will be comprehensively characterized using X-ray diffraction (XRD), Raman spectroscopy, transmission electron microscopy (TEM), Brunauer–Emmett–Teller (BET) analysis, Fourier-transform infrared spectroscopy (FT-IR), and thermogravimetric analysis (TGA). We anticipate that MnFe_2_O_4_/rGO nanoparticles will exhibit enhanced crystalline structure, morphology, and chemical properties that are conducive to high-performance supercapacitor electrodes. The electrochemical performance of these nanocomposites will be evaluated to validate their suitability for advanced energy storage applications.

## 2 Materials and Methods

### 2.1 Reagents and Chemicals

All chemicals used in this study were of reagent grade and employed without further purification. Graphene powder (200 mesh) and graphene nanopowder (8 nm flakes) were purchased from UniRegion Bio-Tech (Taiwan). Hydrogen peroxide (30%), sodium sulfite (Na_2_SO_3_), and iron(II) chloride tetrahydrate (FeCl_2_·4H_2_O) were obtained from Showa Chemical. 1-Methyl-2-pyrrolidinone was acquired from Alfa Aesar (USA). Sodium hydroxide (NaOH), potassium permanganate (KMnO_4_), and sulfuric acid (H_2_SO_4_) were sourced from Nihon Shiyaku (Japan). Manganese(II) chloride tetrahydrate and ploy(vinylidene fluoride) were purchased from Sigma-Aldrich. Potassium hydroxide (KOH) was obtained from Union Chemical (Taiwan). Graphene oxide (GO) was prepared using Hummer’s method.

### 2.2 Synthesis of GO Powder

Graphene oxide (GO) powder was synthesized from natural graphite powder following a modified Hummers method,^28^ employing potassium permanganate and concentrated sulfuric acid as oxidants. Initially, sulfuric acid was placed in a Florence flask and cooled in an ice bath maintained at 5 °C. Subsequently, graphite powder, NaNO_3_, and KMnO_4_ were added to the flask, stirring the mixture in the ice bath for 90 min. The mixture was then stirred and maintained at a temperature of 40–50 °C for 30 min. Following this, 120 mL of deionized (DI) water was added to the flask to raise the temperature to at least 98 °C,^28^ and the mixture was stirred for an additional 30 min. To terminate the reaction, 9 mL of 30% H_2_O_2_ and 300 mL DI water were added.^10^ The synthesized GO was then filtered and washed with 5% HCl to remove any remaining metal ions, followed by rinsing with DI water to eliminate residual ions and acids. Finally, the GO was placed in a drying oven for further application.

### 2.3 Synthesis of MFG Nanocomposites

**Figure 1:**
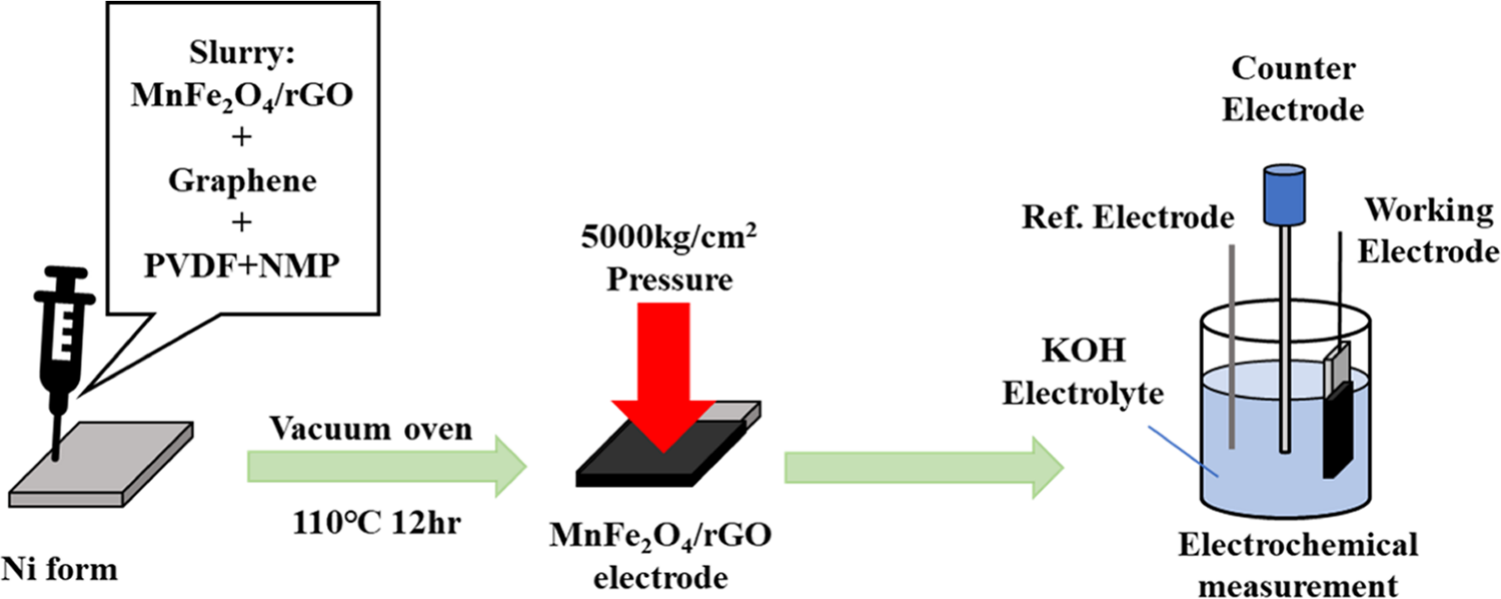
Schematic illustration depicting the formation process of MnFe_2_O_4_/rGO nanocomposites.

MnFe_2_O_4_/graphene oxide (MFG) nanoparticles were synthesized using a microwave hydrothermal method. Initially, 0.05 g GO was initially ground and dispersed in 30 mL DI-water under ultrasonication for 30 min to achieve a homogeneous suspension. Subsequently, FeCl_2_·4H2O and MnCl_2_·4H2O in a molar ratio of 2:1 were mixed with the suspension and sonicated for an additional 30 min. A 2 M solution of NaOH solution was added dropwise to the suspension until the pH reached 12. The synthesis procedure involved transferring the prepared suspension into a Teflon-lined reactor, followed by microwave heating at a power setting of 800 W. This parameter was essential for establishing optimal conditions for the formation of MnFe_2_O_4_/rGO nanocomposites (Figure 1).

The heating temperature was controlled at 160, 180, 200, and 220 °C (labeled as MFG-160, MFG-180, MFG-200, and MFG-220, respectively) with soak times of 5, 10, 30, and 50 min (labeled as MFG-T5, MFG-T10, MFG-T30, and MFG-T50, respectively). Finally, the samples were washed with acetone and DI water, followed by drying and filtration at 75 °C in an oven overnight.

The formation mechanism of this sample involves the electrostatic attraction of positively charged Mn^2+^ and Fe^2+^ ions to the negatively charged GO surface. Under high-temperature conditions, Fe^2+^ is oxidized to Fe^3+^ by the oxygen-containing functional groups on the GO surface, which simultaneously inhibits the oxidation of Mn^2+^.^29,30^ NaOH, acting as a reducing agent, facilitates the precipitation of metal particles onto the rGO.^16^ Furthermore, rGO forms a robust supporting matrix that allows MnFe_2_O_4_ to adhere between the rGO layers.^31^

### 2.4 Preparation of Electrode

**Figure 2:**
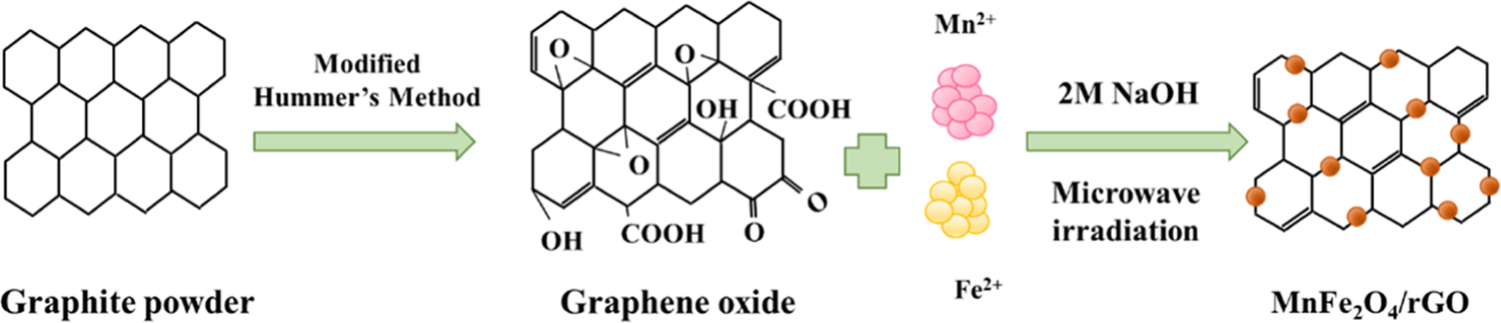
Experimental setup of the Catalyst Instrument.

The active material, carbon black, and poly(vinylidene difluoride) (PVDF) were mixed in a weight ratio of 7:1:2 and combined with a small amount of *N*-methyl-2-pyrrolidone (NMP) to prepare the slurry. This slurry was evenly applied onto a 1 cm^2^ piece of nickel foam using a pipet. The mass of the nickel foam was accurately measured before and after the application of the active material. The foam was then dried and pressed to standardize the mass of the active material to 32.5 ± 2.5 mg. Subsequently, the foam was placed in a vacuum oven and dried for 12 h to produce the working electrode (Figure 2).

### 2.5 Electrochemical Characterization

The electrochemical properties of the prepared electrodes were evaluated using a three-electrode system, with Ag/AgCl serving as the reference electrode and a platinum wire as the counter electrode. Cyclic voltammetry (CV), galvanostatic charge/discharge (GCD), and electrochemical impedance spectroscopy (EIS) were conducted using a CH Instruments CHI760 potentiostat in 6 M KOH electrolyte at room temperature.

### 2.6 Structural Catalyst Characterization

The crystalline structure of the samples was assessed using an X-ray diffraction (XRD, D8A25 eco, BRUKER Co. Ltd., Billerica, MA) with Cu Kα radiation (λ = 1.5418 Å) at 40 kV and 25 mA. A Micro Raman spectrometer (Shamrock 750 spectrograph, Andor Technology, Billerica, MA) with a 567 nm excitation source was used to investigate the vibration modes of the samples, employing a nitrogen-cooled CCD detector. The morphology of the samples was observed using transmission electron microscopy (TEM, Hitachi H-7500, Tokyo, Japan) at 80 kV voltage with an Xe lamp light source. Chemical bonding was investigated using a Fourier-transform infrared spectrometer (FT-IR, JASCO FT/IR-6700, JASCO, Tokyo, Japan) in the wavelength range of 400 to 4000 cm^–1^. Thermo gravimetric analysis (TGA, DT Q600, TA Instruments) was conducted under an N_2_ atmosphere with a heating rate of 10 °C/min from 40 to 800 °C. The N_2_ adsorption–desorption isotherm was investigated using a Brunauer–Emmett–Teller (BET, Nova 800, Anton Paar QuantaTec, Inc.) apparatus at 77 k. Before analysis, the samples were vacuum degassed for 300 min in 120 °C at 10 °C/min. The specific surface and pore size distribution of the samples were analyzed using the BET and Barrett–Joyner–Halenda (BJH) models.

## 3 Characterization

### 3.1 Characterization of MnFe_2_O_4_/rGO

#### 3.1.1 XRD Analysis

**Figure 3:**
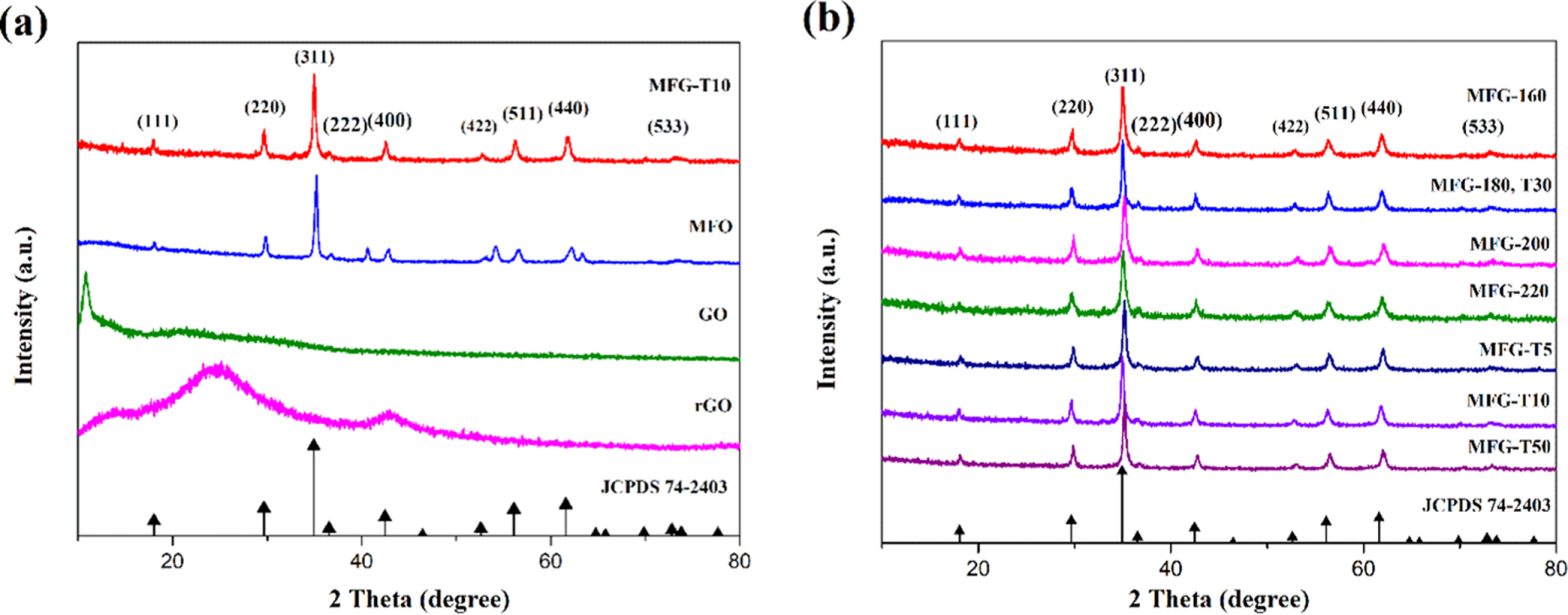
X-ray diffraction (XRD) patterns of (a) MnFe_2_O_4_/rGO, MnFe_2_O_4_, GO, and RGO nanocomposites. (b) XRD patterns of MnFe_2_O_4_/rGO nanocomposites in at various heating temperature and soak times.

Figure 3(a) shows the XRD patterns of GO, rGO, MnFe_2_O_4_, and MnFe_2_O_4_/rGO nanocomposites. Figure 3(b) presents the XRD patterns of MnFe_2_O_4_/rGO nanocomposites synthesized at various heating temperatures and soak times. The XRD pattern of GO displays a strong peak at 2θ = 12.2°, corresponding to the (001) crystal plane. The rGO pattern exhibits two broad peaks at 2θ = 24.2 and 42.7°, corresponding to the (002) and (102) crystal planes, respectively. The patterns of MnFe_2_O_4_ and MnFe_2_O_4_/rGO nanocomposites match the spherical spinel crystal structure. The diffraction peaks observed at 18.3, 30.1, 35.4, 43.1, 53.7, 56.9, 62.5, 72.8, and 73.8° correspond to the (111), (220), (311), (400), (422), (511), (440), (532), and (622) crystal planes of cubic spinel MnFe_2_O_4_ (JCPDS 74–2403).^32^ The plane of (311) is indicative of the cubic spinel structure and is essential for confirming the formation of MnFe_2_O_4_.^2^ The MnFe_2_O_4_/rGO nanocomposites exhibit lower intensity peaks than those of pure MnFe_2_O_4_ structures.^2^ Furthermore, no diffraction peaks from GO (2θ = 12.2°) are observed, indicating that the GO is effectively reduced to graphene and the self-restacking of the reduced graphene sheets is well prevented. Additionally, no peaks corresponding to rGO are detected in the XRD analysis of all MFG samples, as MnFe_2_O_4_ acts as a spacer that separates and grows within the rGO layers.^2^ The absence of peaks from Fe_2_O_3_, MnO, and other crystal phases signifies that the crystal phase is pure manganese ferrite and that the composites were successfully prepared.

#### 3.1.2 Raman spectroscopy

**Figure 4:**
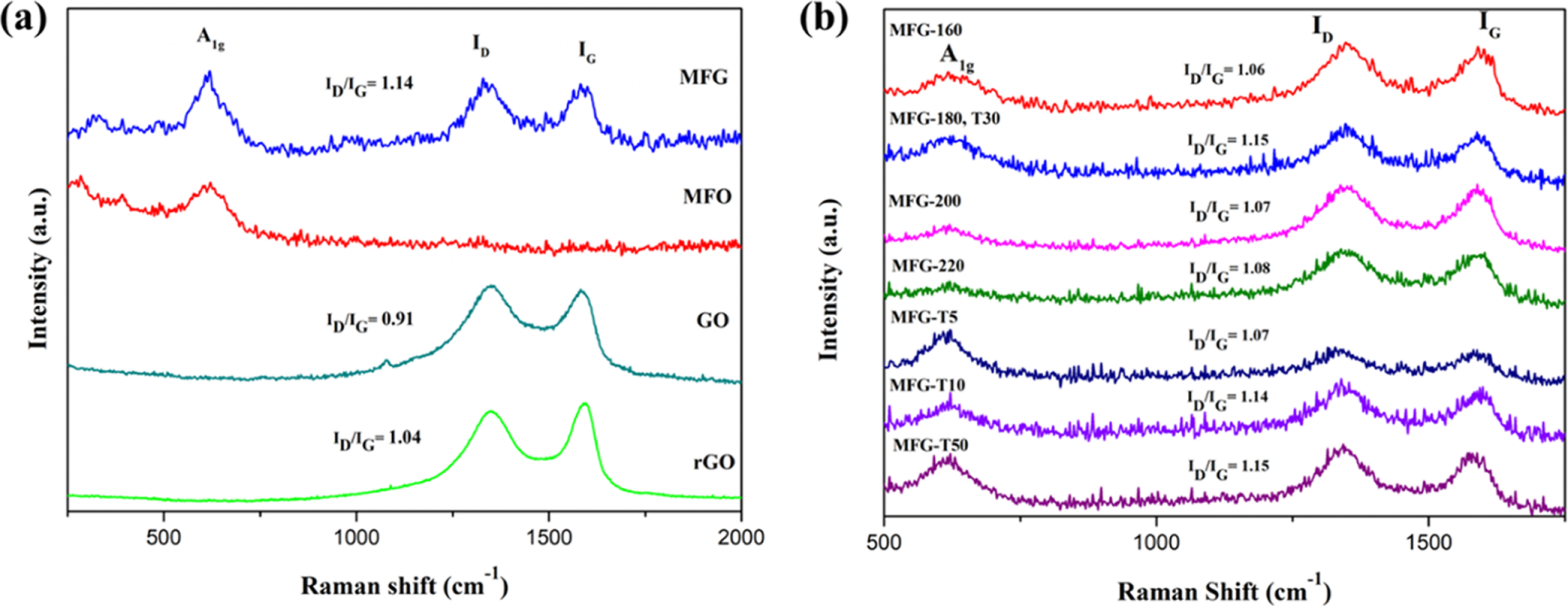
Raman spectra of (a) MnFe_2_O_4_/rGO, MnFe_2_O_4_, GO, and RGO nanocomposites. (b) Raman spectra of MnFe_2_O_4_/rGO nanocomposites at various heating temperature and soak times.

Figure 4(a) presents the Raman spectra of MFG, MFO, rGO, and GO nanocomposites. The GO spectrum displays two significant peaks: the D peak at 1348 cm^–1^ and the G peak at 1580 cm^–1^. The D peak is associated with the vibration of sp^3^ carbon atoms and represents structural defects and disorder in the lattice, while the G peak is related to the vibration of sp^2^ carbon atoms in the two-dimensional (2D) hexagonal lattice.^33,34^ The intensity ratio of the D peak to the G peak (*I*_D_/*I*_G_) is used to evaluate the structural defects, number, and quality of the graphene layers.^35^ For rGO, the D and G peaks appear at 1349 and 1594 cm^–1^, respectively, while for MFG, at 1351 and 1584 cm^–1^. Compared to the GO peaks, the rGO and MFG peaks exhibit a red shift, indicating C atom compensation in the hexagonal network. The *I*_D_/*I*_G_ ratio for GO is 0.91, and after the microwave hydrothermal method, the *I*_D_/*I*_G_ ratio for both rGO and MFG increases to 1.04 and 1.14, respectively. This increase indicates that GO is successfully reduced to rGO during the formation of the nanocomposites. The MFO spectrum shows a characteristic peak at 615 cm^–1^, corresponding to the stretching mode of A_1g_ from MnFe_2_O_4_.^2^ At the A_1g_ mode, MnFe_2_O_4_ exhibits five Raman vibrational modes,^36^ with primary peaks in the range of 618–628 cm^–1^, alongside additional vibrational–rotational modes at 545, 454–463, 321–327, and 175 cm^–1^. The *I*_D_/*I*_G_ ratio for our samples indicates effective reduction of GO to rGO, supporting our findings regarding the structural integrity and electrochemical performance of the nanocomposites. Figure 4(b) shows the Raman spectra of MFG samples synthesized under different parameters, exhibiting both the metal characteristic peak and the graphite characteristic peaks of the D band and G band. Furthermore, the ratio of these graphite characteristics is positively correlated with the degree of reduction.

The ratio of the D band to the G band can be used to estimate the in-plane crystallite size (*L*_a_), calculated using the following eq 1(37)
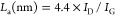
1

Crystallite size is considered an indicator of crystallinity,^38^ and larger crystallite sizes contribute to higher battery capacity.^39^ In our synthesis conducted at different temperatures, we observed an *I*_D_/*I*_G_ ratio of 1.15 at 180 °C, indicating optimal conditions for composite integrity. By maintaining a constant temperature of 180 °C and systematically varying the holding time, we observed an increase in the *I*_D_/*I*_G_ ratio from 1.07 to 1.14 at a holding time of 10 min. Subsequent extensions of the holding time produced only marginal increases in the *I*_D_/*I*_G_ ratio, which ultimately plateaued at 1.15. This finding indicates that a combination of 180 °C and a holding time of 10 min provides the optimal energy conditions necessary for the complete synthesis of MFG nanocomposites.

#### 3.1.3 Morphological Characterization

**Figure 5:**
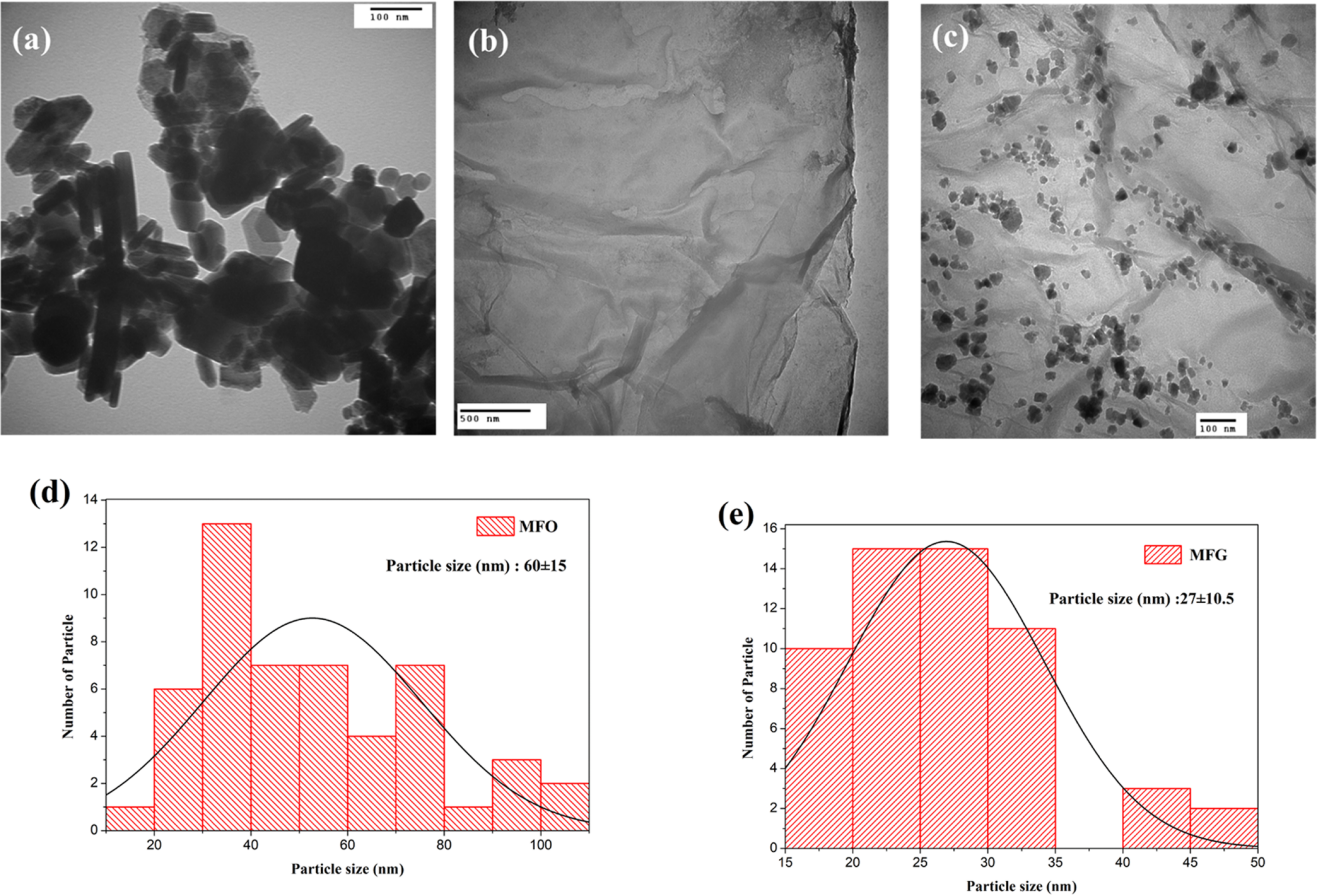
Morphological and structural characterization. TEM images of (a) MnFe_2_O_4_, (b) GO, (c) MnFe_2_O_4_/rGO, and histograms of particle size distribution for (d) MnFe_2_O_4_ and (e) MnFe_2_O_4_/rGO.

The morphology of the nanocomposite was characterized using TEM. Figure 5(a–c) show the TEM images of MnFe_2_O_4_, GO, and MnFe_2_O_4_/rGO, respectively. MnFe_2_O_4_ particles appear spherical and rectangular, forming agglomerates with an average particle diameter of 60 ± 15 nm, as shown in the particle size distribution histogram in Figure 5(d). This aggregation can be attributed to the magnetic interactions between MnFe_2_O_4_ particle.^39^ The GO image reveals a flat, thin, penetrable layer. The MnFe_2_O_4_/rGO image shows that nanocrystals are distributed on the rGO surface, with an average particle size of 40 ± 12.5 nm, as shown in the histogram in Figure 5(e). The addition of rGO modifies the nanoparticles, reducing their size and decreasing their tendency to reunite, which can be attributed to the colloidal system, thus achieving lower energy levels.^40^ The interaction between the metal and the graphene layer at the interface suggests that rGO serves as an effective substrate for the nucleation and growth of metal particles.^2^

#### 3.1.4 FT-IR Spectroscopy

**Figure 6:**
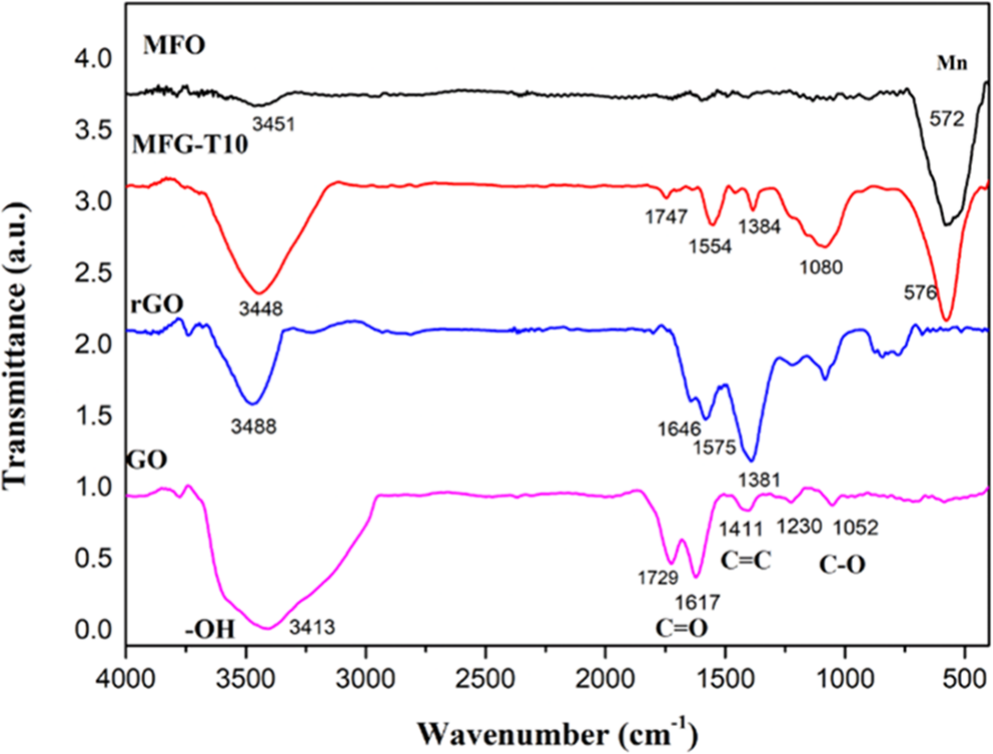
FT-IR spectra of GO, rGO, MFO, and MnFe_2_O_4_/rGO (MFG-T10) nanocomposites.

Figure 6 shows the FT-IR spectrum of GO, rGO, MFO, and MnFe_2_O_4_/rGO (MFG-T10) nanocomposites. Several characteristic peaks are evident in the FT-IR spectrum, indicating the presence of various functional groups. GO exhibits a broad peak at 3413 cm^–1^, which is attributed to the O–H stretching vibration from absorbed water molecules. Peaks around 1729 and 1617 cm^–1^ indicate the presence of carboxylic (C=O) and carboxyl (COOH) functional groups, respectively. The peak at 1411 cm^–1^ results from the aromatic carbon(C=C) stretching vibration. The C–O vibrations from alkoxy and epoxide groups (C–O–C) can be observed at 1052 and 1230 cm^–1^, respectively.^41^ The FT-IR spectra of rGO lack the typical peak of the carboxylic group and show decreased peaks at 1646, 1575, and 1646 cm^–1^, confirming the successful reduction of GO to rGO and validating the Raman results. The MFO spectra exhibits a strong absorption peak of 572 cm^–1^, which is attributed to lattice M–O (M=Fe and Mn) stretching.^42^ In the MFG spectrum, the lattice peak is observed at 576 cm^–1^, and C=C stretching vibration is present at 1384 and 1554 cm^–1^. These observations confirm the successful synthesis of MFG nanocomposites.

#### 3.1.5 TGA Analysis

**Figure 7:**
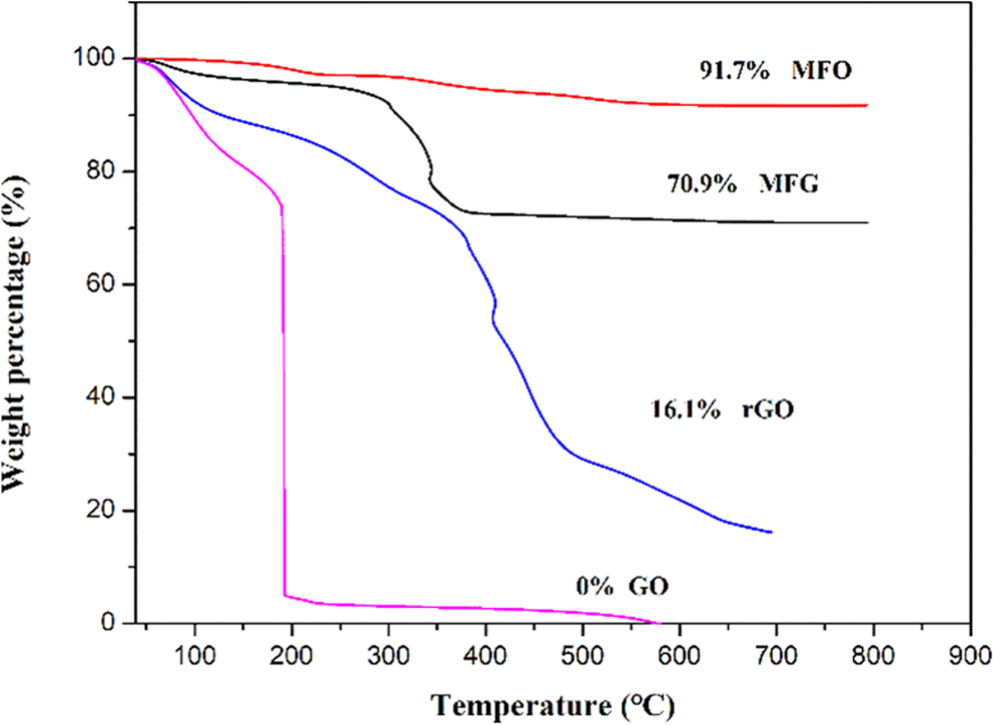
Thermogravimetric analysis (TGA) of GO, rGO, MFO, and MnFe2O4/rGO (MFG-T10) nanocomposites.

Figure 7 shows the TGA analysis of MFO, MFG, rGO, and GO samples. The first weight loss step in the temperature range of 80–200 °C is related to the absorbed water on the surface of GO,^43^ accounting for approximately 10 wt %. The second weight loss step in the temperature range of 300–500 °C is associated with the decomposition of oxygen-containing functional groups in rGO.^40^ Additionally, the GO curve shows approximately 0 wt % after 200 °C due to the combustion of carbon atoms in the graphene sheets with the oxygen during the process.^43^ The TGA analysis of rGO exhibits a slower weight loss rate than GO in the second weight loss step, attributed to the reduction of oxygenic groups during the microwave hydrothermal treatment, which imparts high-temperature stability to rGO.^44^ Moreover, the MFG sample, prepared from 22.8 wt % GO, shows a total carbon weight loss of 20.8 wt %. After complete combustion of rGO at temperatures above 700 °C, MFG retains MnFe_2_O_4_ with a weight of 70.9%.

#### 3.1.6 BET Analysis

**Figure 8:**
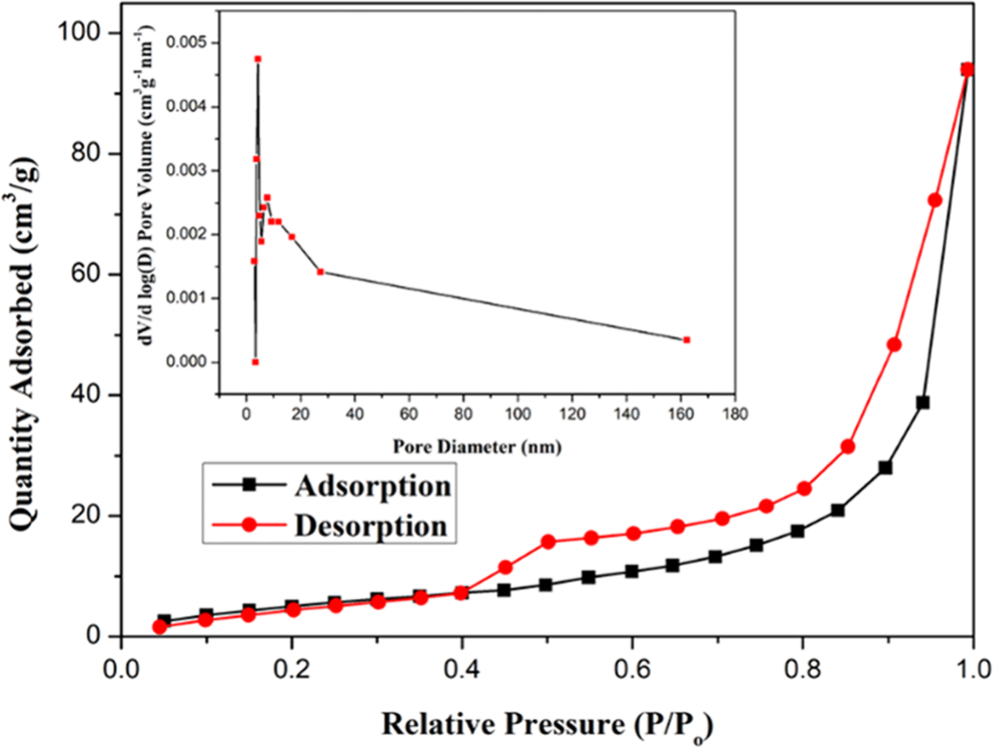
Nitrogen absorption–desorption isotherm and pore size distribution calculated using BJH model of MFG-180, T10 nanocomposite.

Figure 8 shows the N_2_ adsorption–desorption isotherm of the MFG-T10 nanocomposite, revealing its pore characteristics and mesoporous nature. According to the IUPAC classification, the isotherm exhibited a Type IV profile with an H3 hysteresis loop, characterized by a sharp peak in the high-pressure region (*P*/*P*_o_ = 0.8 to 1).^45^ The nanocomposite demonstrates microporosity (2–50 nm), with pores not fully filled with gas during the adsorption–desorption process. The BJH pore size analysis (inset in Figure 8) indicates a pore size of 4.29 nm and an individual pore volume of 0.14 cm^3^/g, respectively. The BET surface area is measured at 37.019 m^2^/g, primarily attributed to the adding of rGO.^2^ A large surface area in the electrolyte can enhance ion penetration, thereby increasing capacitance efficiency. Comparative BET measurements at various synthesis durations (Figure S1) indicate that MFG-T10 exhibits the highest specific surface area (Table S1). As reported in the literature,^3,41^ a larger specific surface area promotes enhanced ion penetration within the electrolyte, increases the availability of active sites during electrochemical testing, and is anticipated to yield improved specific capacitance.

### 3.2 Electrochemical Measurements

#### 3.2.1 Cyclic Voltammetry

**Figure 9:**
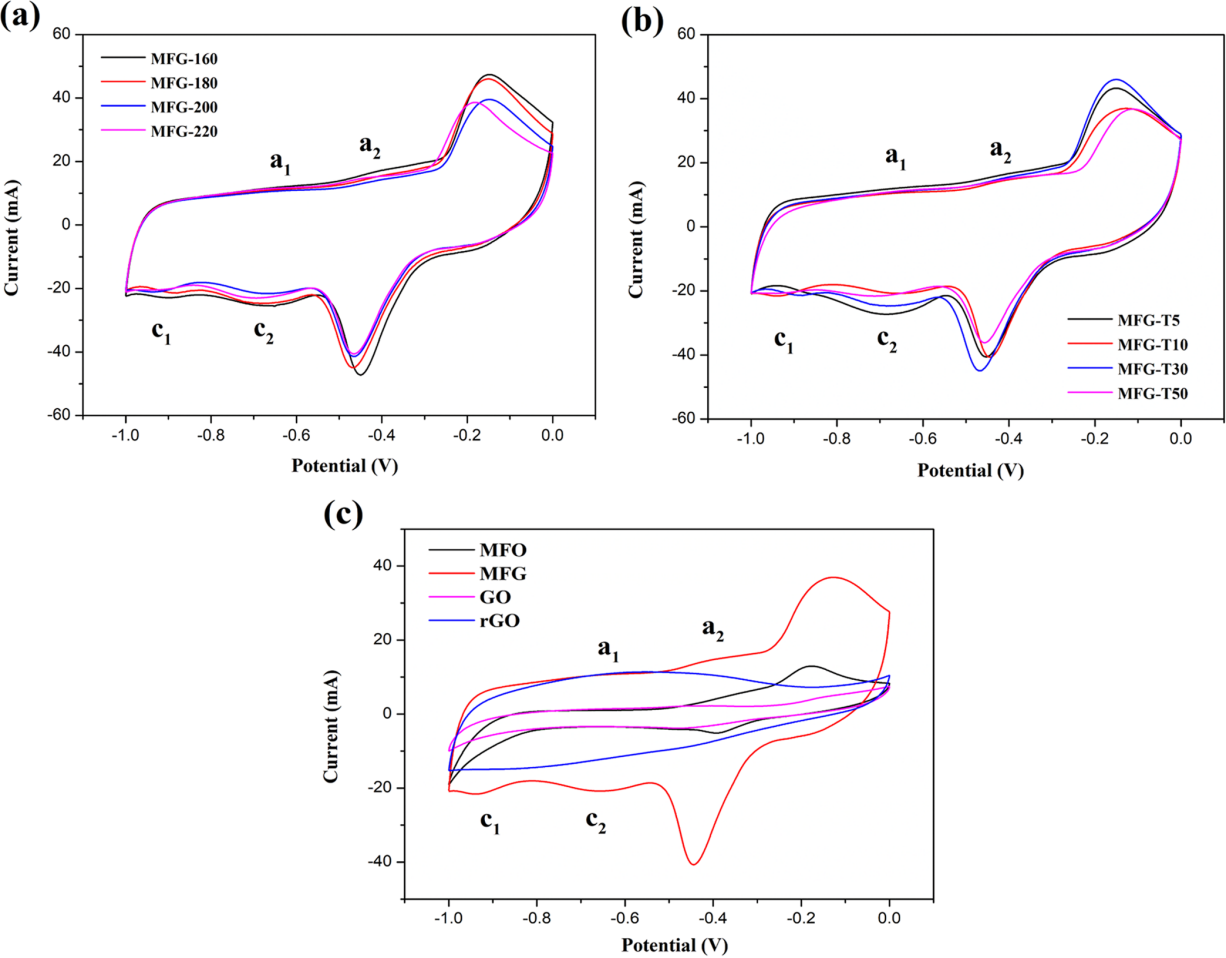
Electrocatalytic performance of (a) different temperatures of MFG (MFG-160 to MFG-220), (b) different parameters of holding time (MFG-T5 to MFG-T50), and (c) MFG, MFO, rGO, and GO.

Electrochemical measurements were measured in a 6 M KOH solution at room temperature. Figure 9(a–c) display the CV curves of MFG under different parameters (MFG-160 to MFG-220, and MFG-T5 to MFG-T50), and rGO, GO, and MFO within the potential range of −1.0–0.0 V. The specific capacitance (*C*_s_) obtained from the CV curves was calculated at a scan rate of 5 mV/s, as shown in Table 1, using the following Formula 2(16,46)
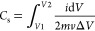
2Where “*i*” is the current (A), ‘“*m*” indicates the mass of the active material (g), “ν” is the scan rate (V/s), and “Δ*V*” is the potential range of measurement. Among the MFG samples synthesized at different temperatures (MFG-160 to MFG-220), MFG-180 exhibited the highest *C*_s_. Subsequently, by using 180 °C for different holding times (MFG-T5 to MFG-T50), *C*_s_ was measured. The optimal parameter was found to be MFG-T10, which showed the highest *C*_s_. All the CV curves of MFG show two pairs of sharp redox peaks indicating that the electrode’s behavior is mainly controlled by pseudocapacitance through a faradaic redox reaction, demonstrating excellent reversibility during charging and discharging.^47^ We propose that MFO and MFG exhibit two types of redox reactions at negative potentials. The first type involves the redox reaction of MnFe_2_O_4_ (eqs 3 and 4),^48,49^ while the second type involves the redox reaction of Fe (eqs 5 and 6)^9,50^
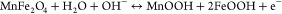
3
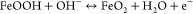
4
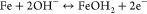
5
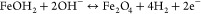
6According to the literature,^9^ the peaks a_1_ and a_2_ observed in the anodic scan can be attributed to the oxidation of Fe to Fe^2+^ and the oxidation of Fe^2+^ to Fe^3+^, respectively. These peaks correspond to the cathodic peaks c_1_ and c_2_, where c_1_ and c_2_ are attributed to the reduction of Fe^2+^ to Fe and the reduction of Fe^3+^ to Fe^2+^, respectively.

**Table 1:** Supercapacitor Parameters of Electrode Material

electrode	specific capacitance (F/g) at 0.005 V/s	discharging time (s)	gravimetric capacitance (F/g)
MFG-160	158.315	375.9	187.95
MFG-180, T30	157.859	377.086	188.543
MFG-200	151.409	343.916	171.958
MFG-220	133.237	305.924	152.962
MFG-T5	152.014	351.914	175.957
MFG-T10	150.581	393.226	196.613
MFG-T50	134.796	314.72	157.36
rGO	73.639	171.358	85.679
MFO	28.021	187.154	93.57
GO	20.441	40.102	20.05
MFG-160	158.315	375.9	187.95

The CV curves of MFO also display similar redox peaks, attributed to the reduction of Mn or Fe. Notably, all the CV curves of MFG show the highest integrated area among those of GO, rGO, and MFO, indicating superior energy-storage performance.

These results suggest that introducing rGO enhances capacitance behavior and improves specific capacitance.

#### 3.2.2 Galvanostatic Charge/Discharge

**Figure 10:**
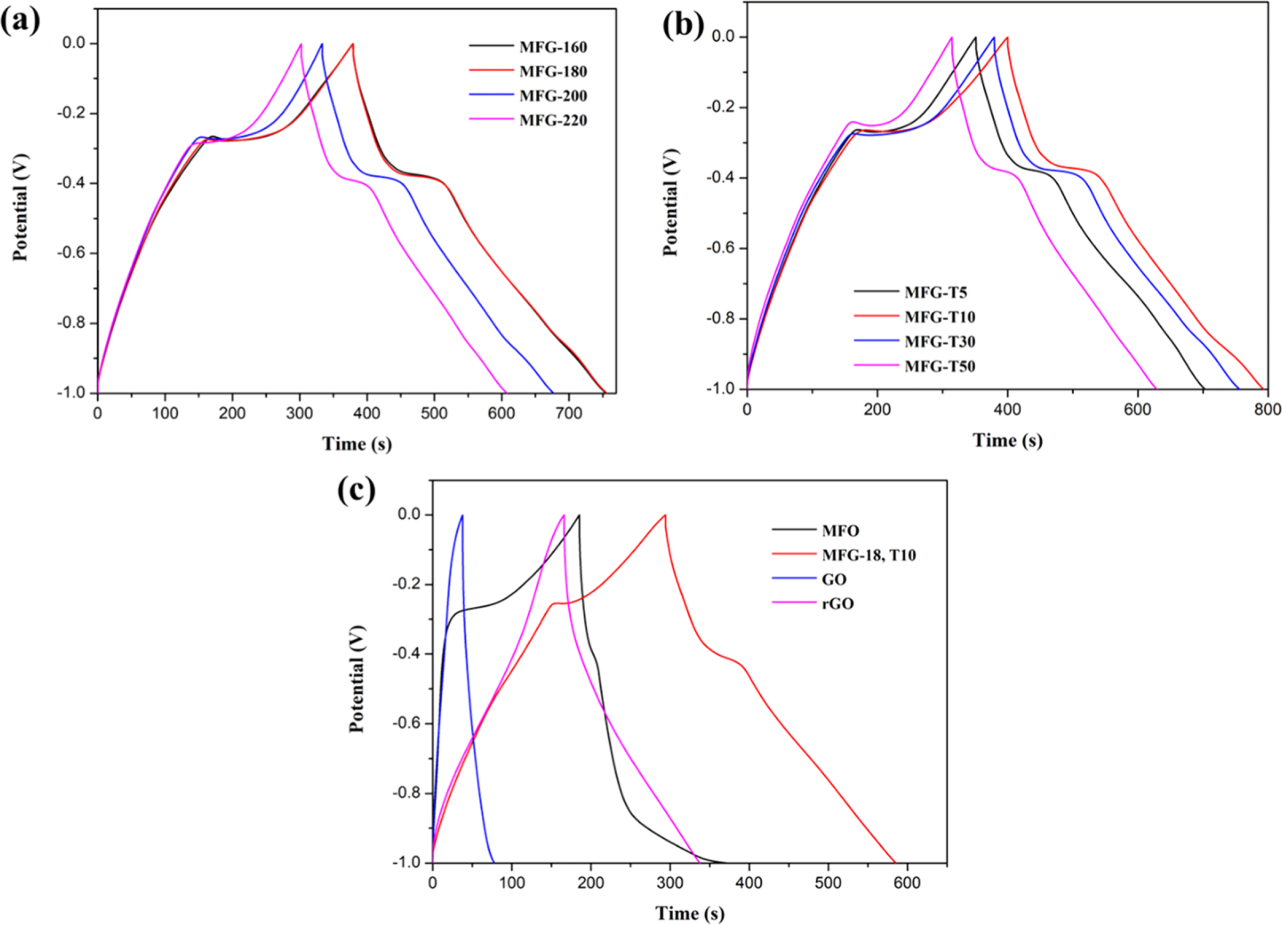
Electrocatalytic performance of (a) different temperatures of MFG (MFG-160 to MFG-220), (b) different parameters of holding time (MFG-T5 to MFG-T50), and (c) comparison of MFG, MFO, rGO, and GO.

Figure 10(a–c) show the GCD curves, performed in the potential range of −1–0 V, to evaluate the storage capability of the electrodes. The specific capacitance (*C*_s_) was calculated from the GCD curves at a scan rate of 5 mV/s, as shown in Table 2, using the following Formula 7(16)
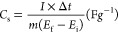
7Where “*I*” is the current density (A/g), “Δ*t*” indicates the discharge time in seconds (s), “*m*” is the active material mass (g), and ‘“*E*_f_ – *E*_i_” indicates the potential range of measurement during charge–discharge. The *C*_s_ values from GCD correspond to the values obtained from CV. According to the equation, a longer discharge time represents a higher specific capacitance, with the optimal parameters identified as MFG-180 and T10.

**Table 2:** Electrochemical Performance of MnFe_2_O_4_ Based Materials for Supercapacitor in Previous Works^a^

sample	preparation method (temperature, time)	electrolyte	Cs (F/g)	capacitance retention	references
MnFe_2_O_4_/rGO	Mic. (180 °C 10 min)	6.0 M KOH	196.613 (0.5 A/g)	90.22% after 6000 cycles	this work
MnFe_2_O_4_/rGO	Tra. (200 °C 24 h)	6.0 M KOH	134.4 (5 A/g)	nearly 80% after 1000 cycles	(41)
MnFe_2_O_4_	Tra. (80 °C, evaporate the solution)	3.5 M KOH	173 (1 A/g)	105% after 10,000 cycles	(15)
MnFe_2_O_4_	Tra. (140 °C 12 h), Annea. (400 °C 2 h)	2 M KOH	282.4 (0.5 A/g)	85.8% after 2000 cycles	(53)
MnFe_2_O_4_ hallow sphere/rGO	Tra. (200 °C 22 h)	3 M KOH + 0.1 M K_4_[Fe (CN)_6_]	768 (8 A/g)	95% after 4000 cycles	(54)
MnFe_2_O_4_ microspheres	Tra. (200 °C 12 h)	2 M KOH	88.4(0.01 A/g)	69.2% after 2000 cycles	(55)
Co_2_AlO_4_@MnO_2_/Fe_2_O_3_	Tra. (90 °C 6 h + 160 °C 24 h), Annea. (350 °C 2 h)	2 M KOH	99.13 (2 A/g)	92.4% after 5000 cycles	(56)
ZnCo_2_O_4_@MnO_2_/Fe_2_O_3_	Tra. (120 °C 2 h)	1 M KOH	161 (2.5 mA/cm ^2^)	91% after 5000 cycles	(57)
Fe_3_O_4_-rGO	Tra. (190 °C 12 h)	1 M NaSO_3_	182.2 (1.25 A/g)	92.4% after 1000 cycles	(58)
MnO_2_	Mic (40 °C 1 h)	0.5 M Na_2_SO_4_	214 (2 mA/cm ^2^)	<90% after 5000 cycles	(59)
NiCoS_4_/GO	Mic. (700 W 10 min), Tra. (120 °C 8 h)	6 M KOH	126.5 (0.5 A/g)	97.0% after 2000 cycles	(60)
ZnFe_2_O_4_/rGO	Mic. (700 W, 7 min, 2450 MHz)	2 M KOH	628 (1 A/g)	89% after 2500 cycles	(3)
Mn_3_O_4_/rGO	Mic. (150 °C 30 min)	1 M Na_2_SO_4_	165 (0.5 A/g)	380.30% after 1000 cycles	(61)
GN/MnO2	Mic. (75 °C 30 min)	1 mol/L Li_2_SO_4_	244 (0.1 A/g)	94.3% after 500 cycles	(62)
Ni(OH)2	Tra. (200 °C 12 h)	1 M KOH	2814 (3 A/g)	57% after 2000 cycles	(17)
α-Co(OH)_2_/rGO	Tra. (100 °C 2 h)	PVA/KOH	260 (0.5 A/cm ^2^)	99.35% after 2000 cycles	(18)

a Mic.: Microwave hydrothermal method; Tra.: Traditional hydrothermal method; Annea.: annealing.

All GCD curves of MFG and MFO nanocomposites show nonlinear profiles, indicating that the redox reaction occurs with faradaic behavior (Figures 10(a–c)) and is unaffected by the addition of a reduced graphene oxide layer.^47^ Additionally, the increased discharge time in MFG is attributed to the presence of rGO, which enhances the specific surface area, and facilitates the Faradaic reaction.^16^ Increasing surface area facilitates the migration path of the ions, thereby reducing diffusion resistance and leading to an increase in specific capacitance.^16,47^ Among the various synthesis conditions, MFG-T10 exhibited the highest specific surface area, which corresponds to its superior GCD performance. A comparison of the gravimetric capacitance and surface area is presented in Figure S2.

#### 3.2.3 Electrochemical Impedance Spectroscopy

**Figure 11:**
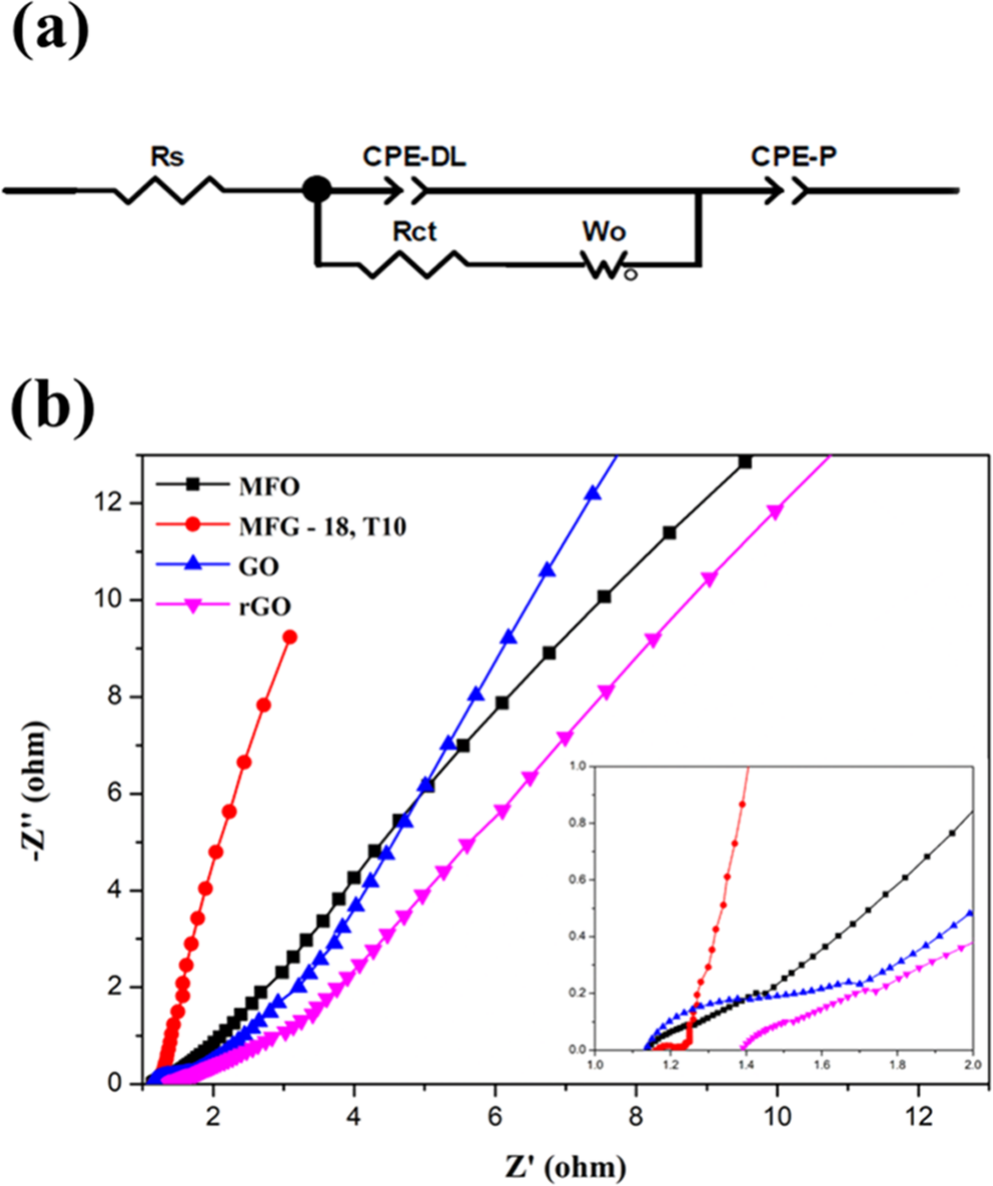
(a) Electrochemical equivalent circuit, (b) the Nyquist plot of MFG, MFO, rGO, and GO.

EIS was employed to evaluate the performance of supercapacitors in terms of charge transfer efficiency, charge impedance, and conductivity. The Nyquist plot was measured within a frequency range of 0.01 to 100 kHz at an amplitude of 0.005 V, and the resistance was fitted using an electrochemical equivalent circuit as shown in Figure 11(a,b) presents the Nyquist plot of MFO, MFG, GO, and rGO, which can be divided into two parts: a semicircle in the high-frequency region and a straight line in the low-frequency region. The high frequency region represents the charge-transfer resistance (*R*_ct_), corresponding to the total resistance at the interface of the electrode and electrolyte.^2^ The diameter of the semicircle is positively correlated with resistance, indicating poorer conductivity with larger diameters. The equivalent series resistance (*R*_s_) is obtained from the intersection of the high-frequency region on the *X*-axis,^41^ corresponding to the total resistance during ionic charge transport in the electrolyte system. The oblique straight line in the low-frequency region indicates ideal capacitive behavior attributed to the rapid and reversible faradic reaction of the electrode material on rGO.^2^ The *R*_s_ values of MFO, GO, and rGO electrodes are calculated as 1.123, 1.17, and 1.454 Ω, respectively. The *R*_ct_ values are 0.451, 0.671, and 0.638 Ω, respectively. However, MFG exhibits a *R*_s_ of 0.901 Ω and a *R*_ct_ of 0.326 Ω, which are the lowest resistance values among the samples. This is further confirmed by the GCD measurements, indicating that MFG has the highest specific capacitance due to its minimal resistance. The low impedance observed can be attributed to several factors, including the larger interlayer spacing of MnFe_2_O_4_/rGO (MFG), as evidenced by Raman spectra, and the smaller particle size observed in TEM analysis. These characteristics contribute to reducing resistance during ion interactions with metal particles, thereby enhancing charge transfer kinetics.

#### 3.2.4 Evaluation of Capacitance Contribution

**Figure 12:**
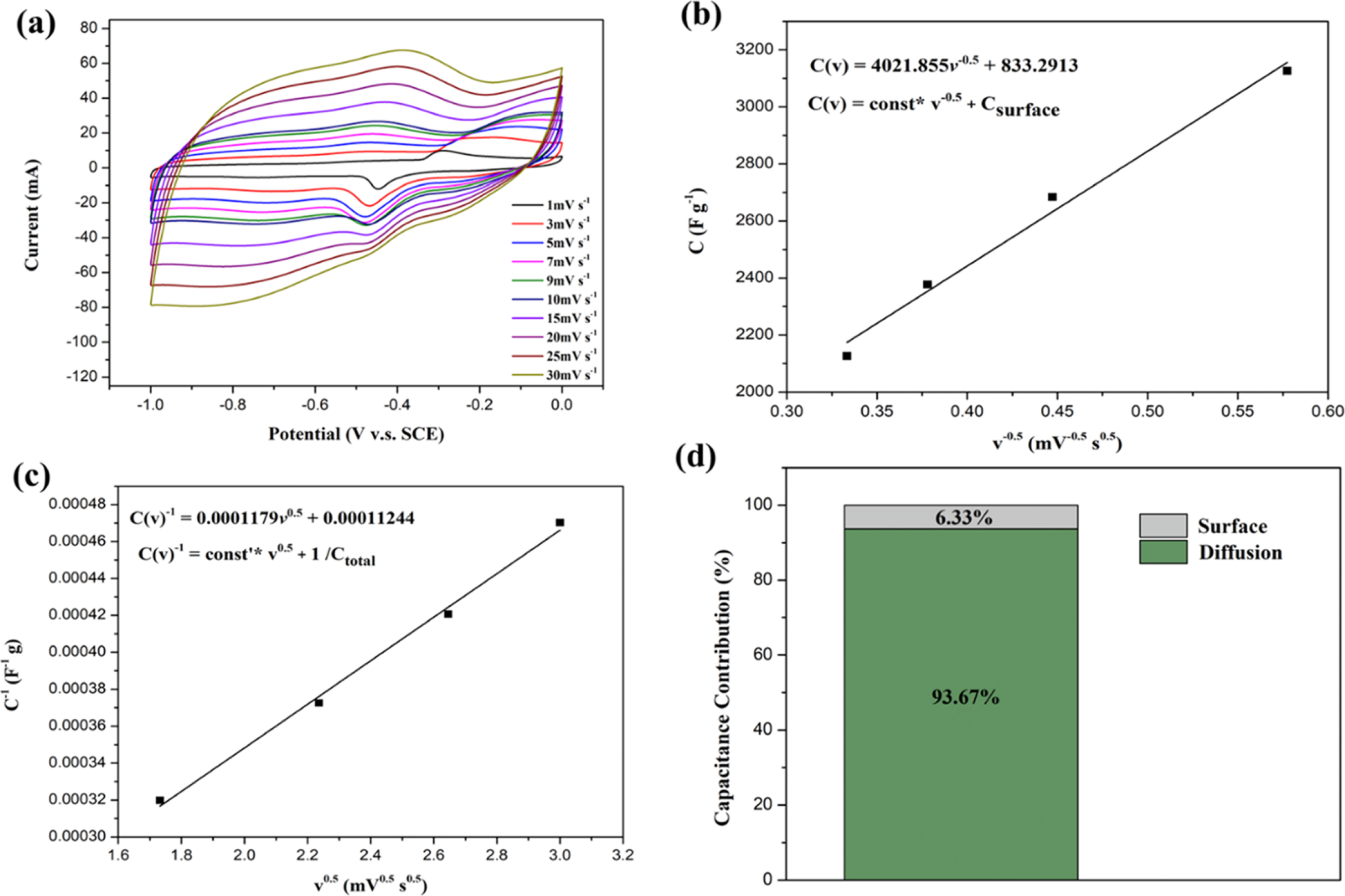
Electrocatalytic performance of MFG (MFG-180, T10): (a) CV curve at various scan rates, (b) Linear fit of gravimetric capacitance versus the reciprocal of the square root of scan rate, (c) Linear fit of specific capacitance versus the square root of scan rate, (d) Contribution analysis of capacitive components for MFG.


The CV curves at various scan rates (1–30 mV/s) of MFG (MFG-180, T10) are shown in Figure 12(a). As the scan rate increases, the oxidation–reduction peaks become more pronounced, particularly the second oxidation peak at −0.5 V. Additionally, the shape of the CV curves remains consistent across different scan rates, indicating good reversibility of the MFG nanocomposite.^47,51^ The fitted slopes of the response peak currents for MFG, MFO, and rGO are presented in Figure S3. The slopes for MFO are 0.21 and 0.12, indicating a diffusion-controlled behavior.^5^ In contrast, the slope for rGO is 0.9, suggesting a predominantly capacitive behavior. This observation can be attributed to the high surface area of rGO, which facilitates ion diffusion and enhances charge storage. For MFG, the slopes are 0.52 and 0.54, indicating that the material’s diffusion behavior lies in the transition region between capacitive and diffusion-controlled processes. The total capacitive behavior is derived from both surface-reaction-controlled and diffusion-controlled processes. To analyze this behavior, the Trasatti method is employed to differentiate the contributions of these processes using the following formulas (8 and 9).^16,51^

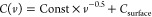
8
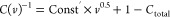
9When *C*, ν, *C*_surface_, and *C*_total_ represent the capacitance, sane rate, surface-reaction-controlled of capacitance and total capacitance. The surface-reaction-controlled capacitance (*C* surface) is determined from the linear fit plot of “*C*^1–^” versus ‘*V*^0.5^’ (Figure 12(b)). Conversely, the total capacitance (*C*_total_) is derived from the plot of ‘*C*’ versus ‘*V*^–0.5^’ (Figure 12(c)). The diffusion-controlled capacitance is then calculated by subtracting *C*_surface_ from *C*_total_. In comparison with ion diffusion behaviors, the diffusion-controlled process and surface-reaction-controlled process represent pseudocapacitance and electrical double-layer capacitance, respectively.^52^Figure 12(d) illustrates the percentage of contribution evaluated for MFG, showing that the primary capacitive behavior is diffusion-controlled, indicative of pseudocapacitance.

#### 3.2.5 Cycling Stability

**Figure 13:**
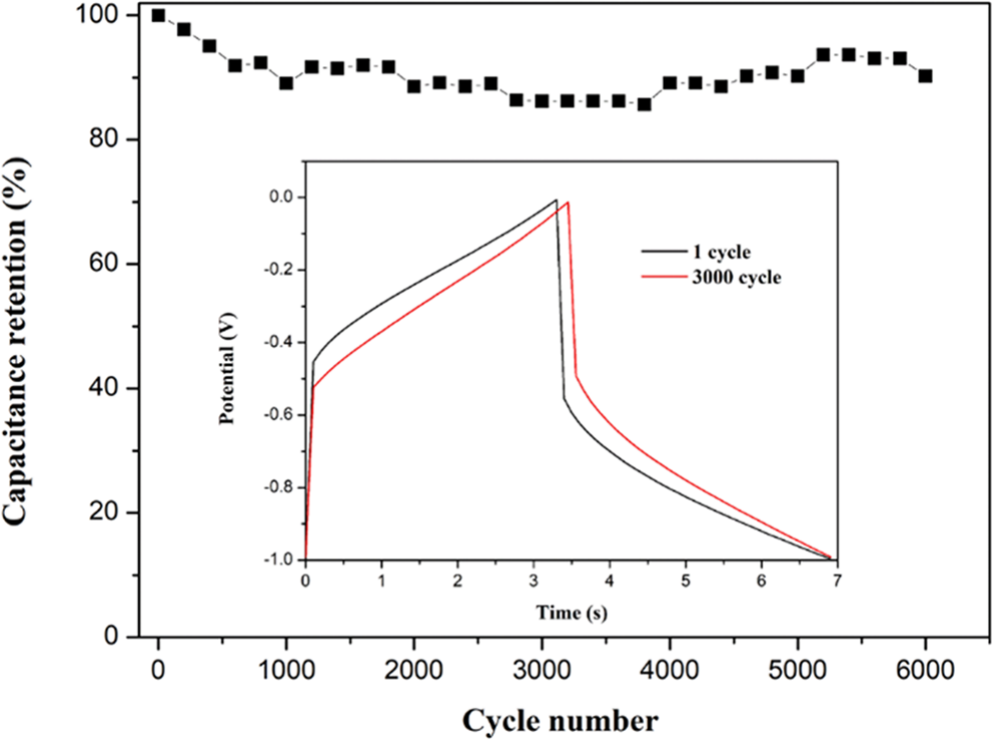
Cyclic stability of MFG over 6000 cycles.

To assess the lone-term cyclic stability of the capacitor, the specific capacitance retention was measured over 6000 GCD cycles at 10 A/g. Figure 13 demonstrates that the capacitance retained 90.22% of its initial value after 6000 cycles. A comparison between the GCD curves of the first and the 6000th cycles showed no significant difference, indicating excellent long-term stability of the capacitor. This high stability and conductivity are attributed to the spinel structure of MnFe_2_O_4_ and the reduced form of graphene, respectively, resulting in high cycling efficiency.^2,16^Table 2 summarizes the performance of MnFe_2_O_4_ and graphene composites in supercapacitors from previous studies, while also comparing the time required for materials prepared through different processes. The MFG-T10 sample synthesized via a one-step microwave-assisted method not only significantly shortens the synthesis time but also substantially enhances the specific capacitance. Additionally, it maintains excellent long-term cycling stability.

## 4 Conclusions

Manganese ferrite (MnFe_2_O_4_/rGO) nanocomposites were successfully synthesized using microwave hydrothermal methods. The nanocomposites synthesized at 180 °C for 10 min demonstrated superior electrochemical performance, achieving a high specific capacitance of 150.581 F/g at a scan rate of 0.005 mV/s in CV measurements. Additionally, in GCD measurements at a current density of 0.5 A/g, the MFG-T10 sample exhibited a capacitance of 196.6 F/g. The long-term cyclic stability of the MnFe_2_O_4_/rGO nanocomposites was particularly notable, retaining 90.22% of their initial capacitance after 6000 cycles at 10 A/g. These impressive electrochemical properties can be attributed to several factors. The small pore size and spinel structure of the rGO surface play a crucial role in enhancing their electrochemical performance, as indicated by BET analysis. The integration of rGO with MnFe_2_O_4_ creates a synergistic effect that improves the conductivity and mechanical stability of the electrodes. rGO serves as an excellent conductive matrix that facilitates efficient charge transfer and reduces the internal resistance of the composite material, as evidenced by EIS. Moreover, the spherical morphology of the MnFe_2_O_4_/rGO nanocomposites, confirmed by TEM, contributes to their high cycling stability and energy density. The uniform distribution of MnFe_2_O_4_ nanoparticles on the rGO sheets ensures a large surface area for electrochemical reactions, enhancing overall capacitance. The microwave hydrothermal synthesis method employed in this study is advantageous due to its simplicity, rapid processing time, and ability to produce high-quality nanocomposites with excellent electrochemical properties.

This study demonstrates that spherical MnFe_2_O_4_/rGO nanocomposites are promising materials for supercapacitor applications. Their high specific capacitance, excellent cyclic stability, and energy density make them suitable for use in energy storage devices. Furthermore, the microwave hydrothermal method provides an efficient and scalable approach for the synthesis of these advanced nanocomposites. Future research could focus on optimizing the synthesis parameters, the ratio of MnFe_2_O_4_ to rGO and exploring the practical applications of these nanocomposites in various energy storage systems.

## Data Availability

All the data are available within the manuscript.

## Supporting Information Available

Nitrogen absorption-desorption isotherm and pore size distribution (Figure S1); BET surface area and BJH pore size measurements (Table S1); comparison of gravimetric capacitance and specific surface area (Figure S2); dependence of parameter b for (a) MFG-T10, (b) MFO, and (c) rGO samples (Figure S3) (PDF)
