# Supplementary material for: One-Step Microwave-Assisted Synthesis of MnFe2O4/rGO Nanocomposites and Their Electrochemical Properties in Supercapacitors
Source: ACS Omega. 2025 Jan 27;10(5):4473–85. doi: 10.1021/acsomega.4c07810 (PMC11822488; doi:10.1021/acsomega.4c07810)
Supplement: Supplementary file 1 — ao4c07810_si_001.pdf [file ao4c07810_si_001.pdf]

One-Step Microwave-Assisted Synthesis of $MnFe_2O_4$/rGO Nanocomposites and their Electrochemical Properties in Supercapacitors

Kun-Yauh Shih* and Hui-Ying Tseng

Department of Applied Chemistry, National Pingtung University, Pingtung County 90003, Taiwan.

* *Email:* **sky@mail.nptu.edu.tw**

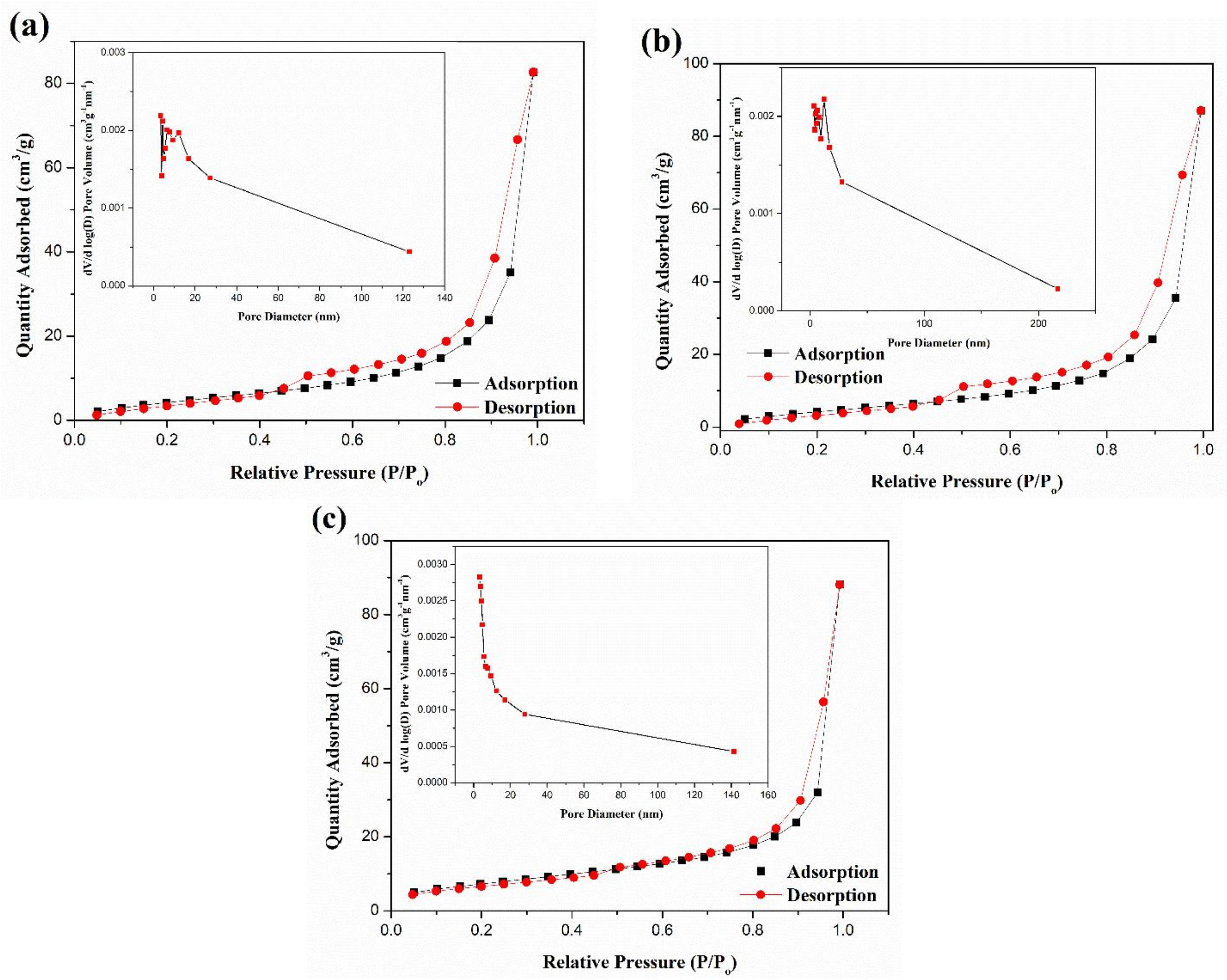

Fig. S1. Nitrogen absorption-desorption isotherm and pore size distribution, calculated using the BJH model, for (a)MFG-T5, (b) MFG-T30, (c) MFG-T50 nanocomposites.

Table. S1. BET surface area and BJH pore size measurements for (a) MFG-T5, (b) MFG-T30, and (c) MFG-T50 nanocomposites.

	Surface Area (m^2/g)	Pore Diameter (nm)	Pore Volume(cm^3/g)
MFG-T5	26.577	3.39	0.12
MFG-T10	37.019	4.29	0.14
MFG-T30	27.797	3.85	0.13
MFG-T50	21.158	3.38	0.13

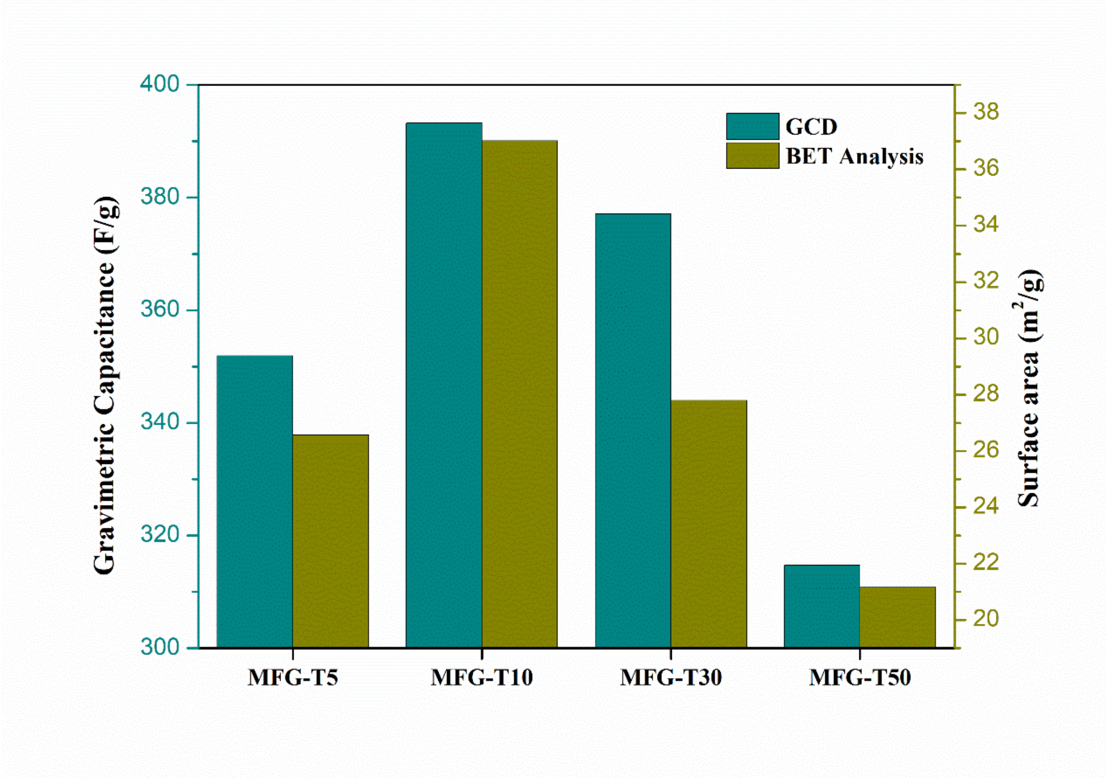

Fig. S2. Comparison of gravimetric capacitance and specific surface area for MFG-T5, MFG-T10, MFG-T30, and MFG-T50 nanocomposites.

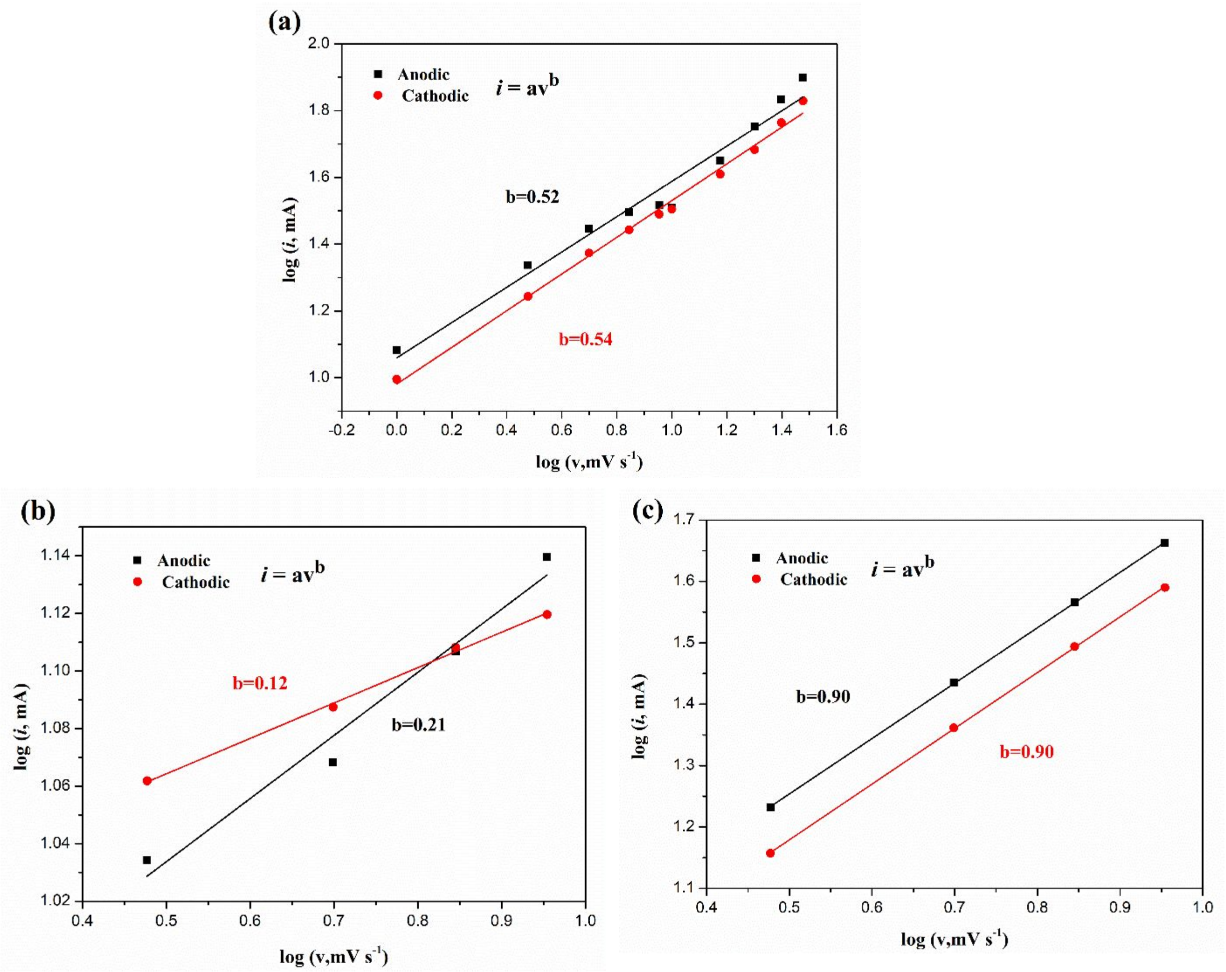

Fig. S3. Dependence of parameter b for (a) MFG-T10, (b) MFO, and (c) rGO samples.
